# Is Acoustic modal analysis a reliable substitution for Osstell® device in dental implant stability assessment? An experimental and finite element analysis study

**DOI:** 10.4317/medoral.26358

**Published:** 2024-01-30

**Authors:** Nima Alimoradi, Mohammadjavad (Matin Einafshar, Reza Amid, Ata Hashemi

**Affiliations:** 1 Department of Biomedical Engineering, Amirkabir University of Technology, Tehran, Iran; 2 Department of Material and Production, Aalborg University, Aalborg, Denmark; 3 Department of Periodontics, School of Dentistry, Shahid Beheshti University of Medical Sciences, Tehran, Iran

## Abstract

**Background:**

Different methods have been proposed to investigate the fixation stability of dental implants, each of which has its limitations. Among these methods, resonance frequency analysis (RFA) has been widely utilized to measure dental implant stability. This study aimed to assess dental implants with two non-destructive RFA and acoustic modal analysis (AMA) validated with a finite element simulation of the fundamental natural frequency (NF) of the bone analog-implant structure.

**Material and Methods:**

A total number of 18 implants were inserted into two Polyurethane (PU) bone blocks with different densities (0.16 g/cc and 0.32 g/cc). AMA was used to measure NF; First, the sound originating from the axial tapping of the implant was recorded with a simple microphone. Secondly, a fast Fourier transformation algorithm was conducted to determine the NF of the implant-bone analog structure. In parallel, the ISQ (Implant Stability Quotient) value was measured using the Osstell® device. Finally, using finite element analysis (FEA), the implant-bone analog structure was modeled for validation.

**Results:**

Doubling the bone analog density resulted in an average increase of 82% and 47% in the NF and ISQ using AMA and Osstell®, respectively (P-value<0.05). Furthermore, a strong linear relationship (R2= 0.93) was observed between the measured NF and ISQ values in the linear regression analysis. The NF of the dental implant predicted by FEA was overestimated by about 15.2% and 15.0% than those in the low- and high-density PUs, respectively. Moreover, the FEA predicted an increase of 83% in NF by increasing the bone analog density from 0.16 to 0.32 g/cc.

**Conclusions:**

Having required the minimum process combined with easily available equipment makes it an ideal method for fixation strength studies. The good correspondence between the ISQ values and NFs, in addition to the good accuracy and reliability of the later method, confirms its application for fixation stability assessment.

** Key words:**Acoustic modal analysis, primary stability, modal analysis, natural frequency, Osstell® test.

## Introduction

Various factors are known to cause the failure of the implantation process, one of which is the lack of proper bonding between the implant and the bone since the bone has not yet fully enclosed the implant (1,2). Another factor is the improper design of the implant which may generate excessive stresses, causing micro-cracks at the bone-implant interface (1-3). Accordingly, the primary and secondary stability of dental implants were defined. Primary stability is the stability of dental implants immediately after implantation and secondary stability is the stability of them after healing and remodeling of adjacent bone (4,5).

Different methods have been developed to assess implant stability. One method is radiography analysis while used along with other assessment techniques (6). Cutting resistance analysis is also another method that measures the energy per volume unit needed for extracting bone; however, it is an invasive procedure (7). Other tests have also been used for fulfilling the process of dental implant stability analysis. Insertion and reverse torque tests have limitations and uncertainties. In particular, irreversible plastic deformation of the implant-bone interface, the failure of the implantation process due to the extra load during the osseointegration stage and the sensitivity of these methods to the stability changes during different processes of interest could lead these methods as destructive methods (8) that cannot be applicable in real operations (9).

The modal analysis method has recently gained interest among researchers mainly due to its good reliability and repeatability among others in studying the fixation strength of the dental implant, orthopedic and spinal bone screws (10-12). The acoustic modal analysis (AMA) is one of the different modal analysis approaches. In this approach, the sound generated by tapping the implant is recorded and then processed by the fast Fourier transformation (FFT) algorithm (11).

Three common examples of the devices fabricated based on modal analysis are the Periotest® device, Osstell® and Implomates® system (13-15). The Periotest® uses an electromagnetic coil and an electric impactor rod in a handheld device. The response generated by the impact is measured by a small accelerometer located at the head of the device. The contact time is calculated in the time domain and then converted to a parameter called Periotest® value, which depends on the damping properties of the tissue around the implant. Lack of sensitivity and resolution has been reported as the limitations of the Periotest® device (16). Osstell® and Implomates® systems have been using resonant frequency analysis (RFA) (3). Osstell® converts the resonant frequency to a scale of ISQ (Implant Stability Quotient) (2). The Implomates® has been extensively studied by Huang *et al*. and Lee *et al*. (17,18) using a small electric rod to generate impact force and stimulate the implant. The response signal in the frequency domain is then transmitted for analysis (2 to 20 kHz). The first peak in the frequency response represents the fundamental resonance frequency of the implant. Kim *et al*. used this stability measurement tool to compare the RFA results obtained by Osstell® (19). They claimed that the peak frequency response is a more sensitive method to density and, hence, provides a better indication of implant stability than the Osstell® system. Assessing the healing process, Gehrke *et al*. reported that the ISQ values increase as the level of osseointegration and respectively bone density increase. It has been reported that this increase is approximately 300 Hz per week (20).

Many researchers have been attempting to extract the natural frequencies of implanTable dental and orthopedic devices in a finite element analysis (FEA) study. Tanimoto *et al*. analyzed the dynamic behavior of a dental implant using three-dimensional finite element modal analysis (3D-FEMA) No experimental validations have been done on their results (21). In another study, nodes of the cylindrical implants and a bone analog were merged at the implant-bone analog interface to model a no-slip condition (i.e., perfect bonding) between the implant and the bone analog block (22). Zaneti *et al*. investigate the influence of implant design on the change in the natural frequency (NF) of the bone-implant system during osseointegration by means of a 3D-FEMA. They have demonstrated the first three NF of bone-implant structure and the mode of vibration in a non-validated study (23).

The described methods have limitations, such as being invasive or costly and inapplicable for all implant models. The RFA methods generally require special devices which could limit their use. Therefore, the goal of the present study was to assess dental implant fixation stability with easily accessible and inexpensive equipment that can be employed for a wide variety of dental implants. The AMA has been used in two different bone analog densities to evaluate the sensitivity of the AMA method to the density. In parallel, a FEM simulation was conducted to validate the fundamental NF of the bone analog-implant structure.

## Material and Methods

- Experiments

**Figure 1:**
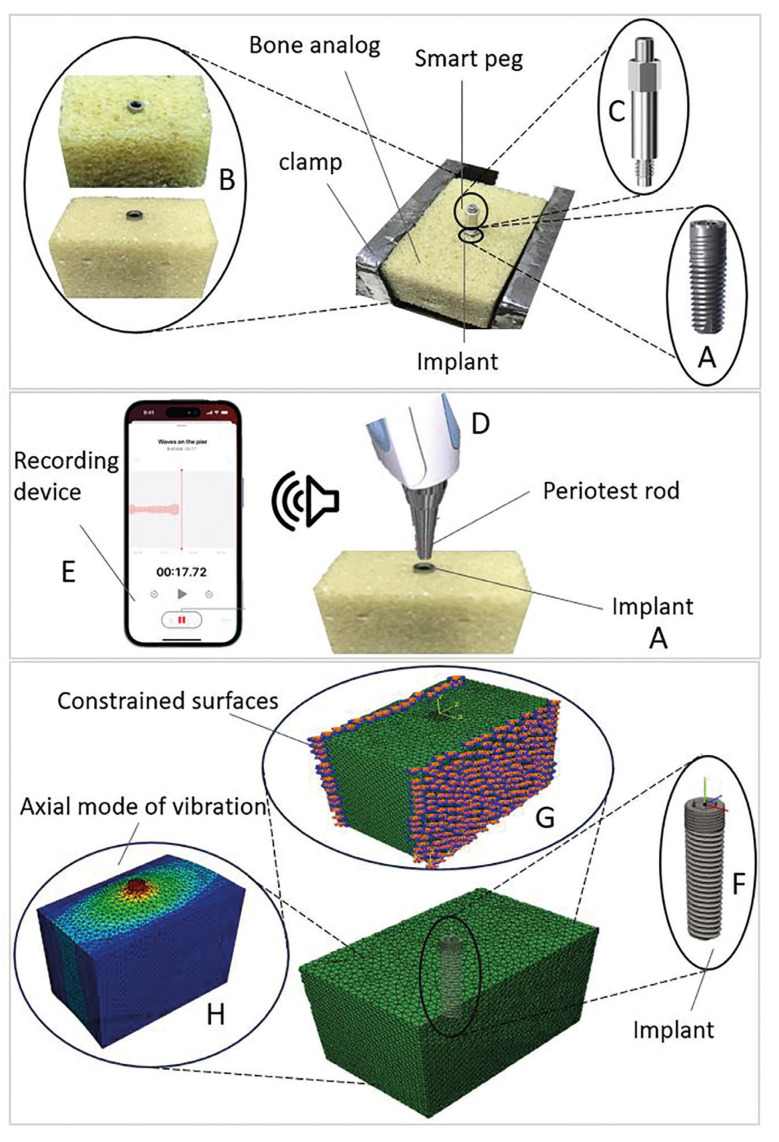
A) Trias dental implant sample for testing purposes was inserted into B) 0.16 g/cc and 0.32 g/cc polyurethane (PU) block and C) the smart peg being mounted on the implant. D) Periotest rod for applying the tapping stimuli, E) sound recording device, F) The 3D model of the conical core and conical thread implant for finite element simulation. G) Two lateral faces were fully constrained and H) The axial mode of vibration extracted from natural frequency extraction simulation.

Sample preparation: Bone analog (Sawbones, Pacific Research Corporation, Vashon, Washington, USA) samples of 40*30*25 mm3 were cut from 40*180*140 mm3 blocks. During the cutting of the samples, a cold-cutting technique was employed by a low-speed cutting machine (BS-1018B, WMT CNC Industrial, China) to ensure no damage to the block structure. The bone analog material blocks, made from rigid polyurethane (PU) foam are commonly utilized as a bone replacement in fixation studies of dental, orthopedic and spinal screws (19). Bone analogs, rather than cadaver bones, were used to provide uniformity and consistency for this comparative study. Blocks with two different densities were used. The low density of 0.16 g/cc had a cell size of 0.5 to 2.0 mm and Young's modulus of 23 MPa, while the higher density of 0.32 g/cc had a cell size of 0.5 to 1.0 mm and Young's modulus of 137.5 MPa, respectively, as a representative of osteoporotic and normal spongy bones (Fig. 1).

Implant insertion: A total number of 18 Trias dental implants (Trias, Servo-dental, Germany) of 10 mm length and 4.5 mm diameter were inserted in PU blocks (N=9/group) (Fig. 1). First, the PU blocks were drilled starting with the small and increasing to the large drill bits, according to the protocol provided by the manufacturer (Cantech, Canada). The pilot hole preparation has remarkable effects on results (24), hence all drilling parameters are kept constant during drilling. Secondly, the implant was inserted into the pilot hole of 4.3 mm diameter and tightened with a torque wrench.

Experimental procedure: After implantation, the stability of the dental implant was evaluated using Osstell® (Osstell® system; Gothenburg, Sweden). In the RFA method, a small electrical converter (SmartPeg) is attached to the implant (Fig. 1) and the frequency is measured by the RFA device. Since the transducer and implant structure are fixed, any change in implant frequency reflects a change in the artificial bone-implant interface bonding. The RFA device quantifies implant stability in the ISQ scale ranging from 0 to 100. To perform the experiments, each sample was tested 4 times in perpendicular directions. After the RFA measurement, a Periotest® rod was used vertically on the top of the implant (Fig. 1). A microphone (Audio-Control CM-145, Apple Inc. USA), with a sampling frequency of 44.6 kHz, was used to record the sound produced from tapping the rod to the head of the implant (Fig. 1). Fig. 1 displays the sample position in the fixture, where both ISQ value measurement and sound recordings were carried out. The FFT analysis of the recorded data was then performed in MATLAB (The MathWorks, Inc. USA) software, and the peak frequency was extracted as the fundamental NF. Afterward, the NFs were compared with the ISQ values. Two groups of high- and low-density PU blocks, each with nine samples, were tested. Furthermore, each test was repeated four times for every sample of both groups.

Statistical analysis: To investigate the effect of density on the NF values a student’s t-test was conducted. Linear regression analysis was used to relate the NF extracted from AMA and ISQ values (Microsoft Excel 2003, Microsoft Corp., Redmond, WA, USA). A confidence level of 95 % (P-value<0.05) was considered to evaluate the statistical differences.

- Simulation

Geometry and Mesh: Three-dimensional (3D) model of the bone analog and the implant (Fig. 1) were created using 3-Matic software (V13, Materialise, Leuven, Belgium) and SOLIDWORKS (Dassault Systèmes, Vélizy-Villacoublay, France), respectively and then transferred into ABAQUS (Dassault Systèmes, Vélizy-Villacoublay, France). The Boolean operation was used to assemble two parts and create the pre-drilled hole in the 3D block model to mimic the experimental setup. The implant was finely meshed using 29503 10-node tetrahedron elements. For the 3D block model, a total number of 22174 10-node tetrahedron elements were used. Mesh convergence analysis was done for different implant-bone analog interface seed sizes of 0.25, 0.5, 1, and 2 mm. The first NF of the first vibrational mode was checked for convergence. At the interface, the meshes of the bone screw matched those of the block material. The mesh independency analysis demonstrated that with a seed size of 0.5 mm convergence analysis was reached; hence, a 0.5 mm seed size around the pilot hole was chosen for all subsequent numerical analyses. The frequency of the first mode was used to determine how well the FE model replicated the experimental test.

Material Properties: Two densities of 0.16 g/cc and 0.32 g/cc, Young’s modulus of 23 MPa and 137.5 MPa, and Poisson’s ratio of 0.3 were assigned to the solid-designed blocks, respectively. The Young’s modulus and density of the implant were assigned 110 GPa and 4.662 g/cc, respectively.

Boundary Conditions: Two lateral surfaces of the PU and PE blocks were fully constrained in three directions i.e., Ux=Uy=Urz=0, replicating the experimental setup test (Fig. 1). A full tie interaction (fully bonded) was assumed to the bone analog-implant interface. A linear perturbation analysis was used to carry out the modal analysis. Geometric non-linearity was considered. The Lancsoz Eigen solver was incorporated to solve the models. The simulations were run on Microsoft Windows (Intel ® Xeon ® Gold 6152, 16 GB RAM) for an average time of 15 minutes per analysis. For further assessment, the relationship between the NF and the block density of 0.12, 20, and 24 g/cc with Young’s modulus of 12.4, 47, and 68 MPa, respectively, were also included in the study.

## Results

- Experimental Modal Analysis

**Figure 2:**
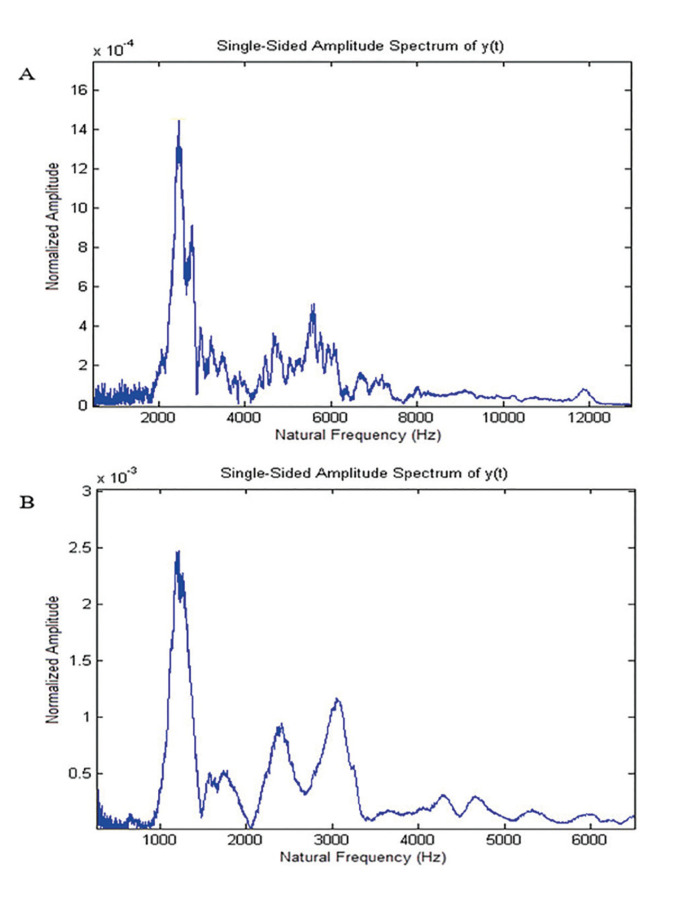
Fast Furrier transform (FFT) plot of sound response in the high-density bone analog of 0.32 g/cc and the low-density bone analog of 0.16 g/cc were measured a) 2465 and b) 1251 Hz, respectively.

Typical FFT analysis of the recorded sound in both high- (0.32 g/cc) and low-density (0.16 g/cc) samples are shown in Fig. 2, respectively. In modal analysis, the first peak with a small width and a high sharpness is considered the fundamental NF.

**Figure 3:**
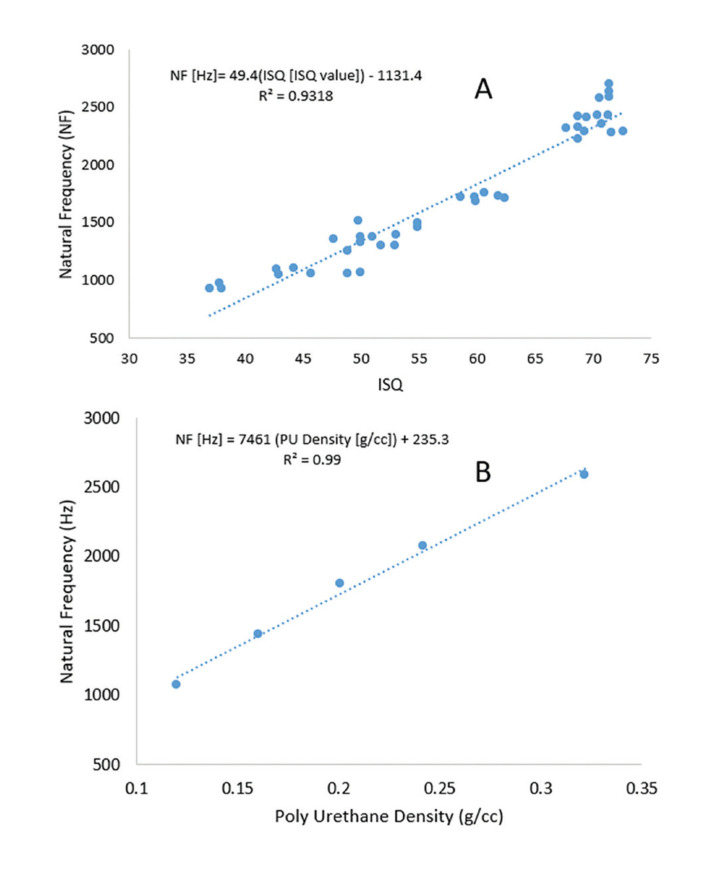
A) Correlation between stabilities as measured by peak natural frequency (NF) and ISQ value for all the samples tested in both block densities. B) Linear prediction of finite element analysis between natural frequency (NF) of implant-bone analog structure vs density of bone analog.

**Figure 4:**
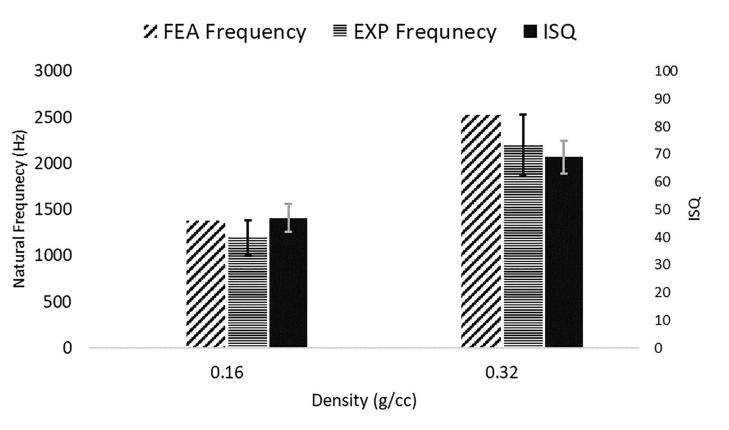
Acoustic modal analysis (AMA) and Osstell® device measurement in bone analog densities of 0.16 and 0.32 g/cc.

A Strong linear relationship between AMA and RFA measurements was established (R2=0.93) and the linear equation of NF=49.4 ISQ-1131.4 was obtained (Fig. 3). The mean NF for the dental implant was 1219±194 Hz and 2239±312 Hz, respectively, in low- and high-density PU blocks (Fig. 4). It is shown that by doubling the block density, i.e., from 0.16 g/cc to 0.32 g/cc, the mean NF was almost doubled. The t-student represented a significant difference between the two groups (P-value<0.001). In the same way, the ISQ values were 47.9±5.26 and 68.4±4.48 in low- and high-density PU blocks, respectively (Fig. 4). An increase in bone analog density also made an increase in the ISQ values. A significant difference between the two groups was observed (P-value<0.001). The results also indicate that the sensitivity of AMA results was higher than that predicted by ISQ values, that is, a rise of 82% for the AMA compared to a rise of 47% for the later method obtained by doubling the bone analog density. This advantage of the modal analysis allows to detection of smaller variations in density and possibly the fixation strength.

- Finite Element Analysis

The FEA was performed to validate the experimental AMA (Fig. 1). To simulate the experimental test setup, FE models were developed. To compare experimental results with those of FEA, the mean NF value of AMA for each density (0.16 g/cc and 0.32 g/cc) was calculated and compared with that of simulation. NFs from the simulation were calculated at 1393 Hz and 2550 Hz in low- and high-density PUs, respectively. Since FE is a powerful tool that can closely predict the NF of the system, therefore, it was used to evaluate the effect of material property alteration on the frequency response. For this part, three extra densities of 0.12, 0.20, and 0.24 g/cc were also included in the study, and the NFs of 1049, 1775, and 2037 Hz were determined, respectively. The linear regression analysis suggested a powerful linear relationship between bone analog density and NF (R2=0.99) and a linear equation of NF= 7461(PU density) +235.3 was obtained (Fig. 3).

## Discussion

The purpose of the current study was to design and define a simple and applicable mechanism of impact and acoustic responses created for the evaluation of dental implant stability using modal analysis. In our study, we used artificial bone blocks with two different densities and porosities. The tapping sound data were recorded and analyzed to calculate NF as the measure of dental implant stability. The results indicated that the peak frequency increases with the rise in the density of the trabecular bone (Fig. 3). Furthermore, the linear regression analysis showed that peak natural frequencies and ISQ numbers are linearly correlated so that any increase or decrease in one of them results in a similar pattern in the other (Fig. 4). Furthermore, the recorded ISQ values within this investigation spanned from 37 to 73. These values effectively demonstrated a spectrum of bone conditions, ranging from unfavorable outcomes to successful results. This variation aptly showcased the capabilities of the RFA device. Baftijari *et al*. showed that there is a strong linear correlation between dental implant stability and ISQ number, as evaluated by RFA (13). A successful implant typically has an ISQ number greater than 65 and less than 50 may indicate potential failure or increased risk of failure (19). The same linear correlation has also been reported by Pan *et al*., indicating that as trabecular bone density increases, the measured NF linearly increases, as well. Also, a strong linear relationship between resonance frequency and ISQ values has been reported in this regard (25).

All 2 groups of interest showed an increase in values of natural frequencies and ISQ. NF increased 82% by varying density from 0.16 g/cc to 0.32 g/cc. In a study by Huang *et al*., a dental implant was fixed in the clamped bone block containing a trabecular and cortical part (26). The experimentally measured peak frequency measured by using an impact hammer was approximately 10 kHz while that predicted by FEA was 17.5 kHz. Given the material used in their study had a much higher density and modulus than that in our study, such a large difference in results could be expected. The boundary and support conditions in their study are such that the implant has more vibration limitations, all of which lead to an increase in the NF (11).

Kim *et al*. measured peak frequency and ISQ number in the same PU blocks (19). They also utilized the dense PU 0.8 g/cc as the cortical bone. The results for the two 0.16 g/cc and 0.32 g/cc samples were reported to be 263.69 Hz and 309.57 Hz and ISQ values ranged from 42.2 to 73, respectively. The mean values ​​obtained for our samples from the experimental tests were 1219 Hz and 2239 Hz, respectively. The reason for this difference can be attributed to the type of tests performed in Kim's study. They used an adapter block which was installed on the implant, and frequency response was obtained after hitting this adapter, while in the present study, the implant itself was impacted axially. As a result, the frequency response in Kim's study was the frequency of the whole system, namely the implant and the adapter assembly, which in turn reduced the system's frequency, due to the added mass of the adaptor. Kastel *et al*. investigated and found no effect of the insertion torque of smart pegs on the ISQ number, and consequently on implant stability (27). Using AMA, the dental implant stability was assessed by varying the clamping force on the implant sample, as a representative of the level of osseointegration. It was shown that more tightening of the clamp increased the NF from 8059.9 Hz to 19086.1 Hz. The clamping torque was reported to change from 2 N.m to 10 N.m (28).

Based on the results of the present study the experimental tests and simulation analyses have a difference of about 14% for both bone analog blocks. As mentioned earlier, in the FEA model a perfect bonding between two material surfaces was considered which was contrary to what happened in the experimental tests. This can explain why the FEA model overestimates the results compared to those of experiments. Also, due to the lack of proper implant stability in the low-density samples, the ISQ number and NF variations, occur over a relatively wide range, and data is scattered as presented in Fig. 3.

The limitations of the study encompass several factors that may impact the applicability of the approach in real-world clinical conditions. One noTable limitation is the use of simplified artificial bone analog blocks with predefined densities and porosities. While these blocks allow for controlled experimentation, they may not fully replicate the intricate complexity and variability inherent in actual human bone. This lack of fidelity to real-world conditions could potentially restrict the generalizability of the findings to diverse bone conditions encountered in clinical practice. Moreover, the finite element analysis (FEA) simulations utilized in the study assumed perfect bonding between material surfaces during impact. This simplification may deviate from actual conditions and lead to a potential overestimation of outcomes in contrast to experimental measurements. This factor could impact the accurate interpretation of relationships between variables. The study primarily focused on the mechanical facets of implant stability evaluation through modal analysis and peak frequency measurements. However, in usual clinical conditions, ambient sounds such as aspirators and compressors, along with the individual anatomical variations in each patient's mouth, may introduce interference and affect the resonance of the sound captured by the microphone. These real-world factors were not explicitly considered in the experimental setup, and their potential influence on the outcomes should be acknowledged. Additionally, the study's scope did not encompass the comprehensive range of factors influencing implant success in clinical practice, such as biological elements (e.g., tissue integration) and diverse loading conditions. It would be valuable to validate the data *in vitro* and *in vivo*, considering the impact of ambient sounds and individual anatomical conditions on the captured sound resonance. This would provide a more holistic understanding of the approach's feasibility in real-world clinical scenarios.

## Conclusions

The main goal was to assess dental implant stability with the minimum and in-hand equipment such as a simple microphone and impactor. We evaluated the dental implant stability by using AMA and extracted the fundamental NF of the implant-block construct. Then, the Osstell® device was used to measure the ISQ numbers as another implant stability measurement tool. The results of sound modal analysis had a good and linear correlation with that of the ISQ numbers. Both methods showed that the reduction of bone density lowered the implant fixation stability. Finally, the finite element method was utilized, and the overall good agreement between its results and those of the experimental allowed further evaluation of other bone densities on the implant stability to be carried out by FE models.

## References

[1] Baggi L, Cappelloni I, Girolamo M (2008). The influence of implant diameter and length on stress distribution of osseointegrated implants related to crestal bone geometry: A three-dimensional finite element analysis. The Journal of Prosthetic Dentistry.

[2] Comuzzi L, Tumedei M, Pontes AE (2020). Primary Stability of Dental Implants in Low-Density (10 and 20 pcf) Polyurethane Foam Blocks: Conical vs Cylindrical Implants. International Journal of Environmental Research and Public Health.

[3] Danza M, Zollino I, Paracchini L (2009). 3D finite element analysis to detect stress distribution: spiral family implants. Journal of Maxillofacial and Oral Surgery.

[4] Gursoytrak B, Ataoglu H (2020). Use of resonance frequency analysis to evaluate the effects of surface properties on the stability of different implants. Clinical Oral Implants Research.

[5] Aragoneses JM, Suárez A, Brugal VA, Gómez M (2019). Frequency Values and Their Relationship With the Diameter of Dental Implants. In: Prospective Study of 559 Implants. Implant Dentistry.

[6] Atsumi M, S-h P, Wang HL (2007). Methods used to assess implant stability: current status. Int J Oral Maxillofac Implants.

[7] Friberg B, Sennerby L, Roos J (1995). Evaluation of bone density using cutting resistance measurements and microradiography. In: An in vitro study in pig ribs. Clin Oral Implants Res.

[8] Einafshar M, Hashemi A, Lenthe GH (2021). Homogenized finite element models can accurately predict screw pull-out in continuum materials, but not in porous materials. J Computer Methods Programs in Biomedicine.

[9] Turkyilmaz I, Tumer C, Ozbek EN, Tözüm TF (2007). Relations between the bone density values from computerized tomography, and implant stability parameters: a clinical study of 230 regular platform implants. Journal of Clinical Periodontology.

[10] Einafshar M, Hashemi A, Lenthe GH (2022). Replacement of Destructive Pull-out Test with Modal Analysis in Primary Fixation Stability Assessment of Spinal Pedicle Screw. Arch Bone Jt Surg.

[11] Einafshar M, Hashemi A, Kiapour A (2022). Evaluation of the efficacy of modal analysis in predicting the pullout strength of fixation bone screws. JOR Spine.

[12] López AB, Martínez JB, Pelayo JL, García CC, Diago MP (2008). Resonance frequency analysis of dental implant stability during the healing period. Med Oral Patol Oral Cir Bucal.

[13] Baftijari D, Benedetti A, Stamatoski A (2019). Influence of Resonance Frequency Analysis (RFA) Measurements for Successful Osseointegration of Dental Implants During the Healing Period and Its Impact on Implant Assessed by Osstell Mentor Device. Open Access Macedonian Journal of Medical Sciences.

[14] Ito FA, Jorge J, Vargas PA, Lopes MA (2009). Histopathological findings of pleomorphic adenomas of the salivary glands. Med Oral Patol Oral Cir Bucal.

[15] López AB, Diago MP, Cortissoz OM, Martínez IM (2006). Resonance frequency analysis after the placement of 133 dental implants. Med Oral Patol Oral Cir Bucal.

[16] Salvi GE, Lang NP (2004). Diagnostic parameters for monitoring peri-implant conditions. Int J Oral Maxillofac Implants.

[17] Huang HM, Chiu CL, Yeh CY (2003). Early detection of implant healing process using resonance frequency analysis. Clinical Oral Implants Research.

[18] Lee SY, Huang HM, Lin CY, Shih YH (2000). In Vivo and In Vitro Natural Frequency Analysis of Periodontal Conditions: An Innovative Method. Journal of Periodontology.

[19] Kim DS, Lee WJ, Choi SC (2014). Comparison of dental implant stabilities by impact response and resonance frequencies using artificial bone. Med Eng Phys.

[20] Gehrke SA, Marin GW (2015). Biomechanical evaluation of dental implants with three different designs: Removal torque and resonance frequency analysis in rabbits. Ann Anat.

[21] Tanimoto Y, Hayakawa T, Nemoto K (2006). Mode superposition transient dynamic analysis for dental implants with stress-absorbing elements: a finite element analysis. Dental materials.

[22] Hernandez BA, Freitas JP, Capello Sousa EA (2023). Fatigue life estimation of dental implants using a combination of the finite element method and traditional fatigue criteria. Proc Inst Mech Eng H.

[23] Zanetti EM, Ciaramella S, Calì M (2018). Modal analysis for implant stability assessment: Sensitivity of this methodology for different implant designs. Dent Mater.

[24] Einafshar M, Shahrezaee M, Shahrezaee MH, Sharifzadeh S (2020). Biomechanical Evaluation of Temperature Rising and Applied Force in Controlled Cortical Bone Drilling: an Animal in Vitro Study. Jt Surg.

[25] Pan CY, Liu PH, Tseng YC (2019). Effects of cortical bone thickness and trabecular bone density on primary stability of orthodontic mini-implants. Journal of Dental Sciences.

[26] Huang HM, Lee SY, Yeh CY, Lin CT (2002). Resonance frequency assessment of dental implant stability with various bone qualities: a numerical approach. Clinical Oral Implants Research.

[27] Kästel I, Quincey G, Neugebauer J (2019). Does the manual insertion torque of smartpegs affect the outcome of implant stability quotients (ISQ) during resonance frequency analysis (RFA)?. Int J Implant Dent.

[28] Huang HM, Pan LC, Lee SY (2000). Assessing the implant/bone interface by using natural frequency analysis. Oral Surg Oral Med Oral Pathol Oral Radiol Endod.

